# Pneumonia: A Cross-Sectional Study of the Quality and Reliability of the Content on Instagram

**DOI:** 10.7759/cureus.45156

**Published:** 2023-09-13

**Authors:** Ihsan Sidhu, Ashik Ali, Abdul Aziz Mohammed, Sravani Kommuru, Eryka Roxas, Javlon Makhkamboev

**Affiliations:** 1 Internal Medicine, King Edward Medical University, Lahore, PAK; 2 Paediatrics and Child Health, SRM (Sri Ramaswamy Memorial) Medical College, Chengalpattu, IND; 3 Internal Medicine, Kamineni Institute of Medical Sciences, Hyderabad, IND; 4 Medicine, Dr. Pinnamaneni Siddhartha Institute of Medical Sciences & Research Foundation, Vijayawada, IND; 5 Internal Medicine, Cebu Institute of Medicine, Cebu City, PHL; 6 Medicine/Internal Medicine, Tashkent Pediatric Medical Institute, Tashkent, UZB

**Keywords:** covid-19, cross-sectional observational study, quality and reliability scores, instagram, pneumonia

## Abstract

Introduction: In today's society, Instagram (Meta Platforms, Inc., Menlo Park, California, United States) has grown to be a platform of enormous importance. It has completely changed the way we connect, share, and consume content, with several active users. Instagram may be a powerful tool for education, helping to inform and raise public awareness of a range of health conditions, including pneumonia. The present paper aims to evaluate and analyze the type, quality, and reliability of information about pneumonia being shared on Instagram.

Methodology: Using the hashtags * #pneumonia, #pneumoniasucks, #pneumoniatreatment, #pneumoniaawareness, #pneumoniaviral, #pneumoniaisnojoke, *data regarding the type of post, number of audience reached, and type of uploader was collected from the related Instagram posts. Global Quality Scale (GQS) and DISCERN scores were used to analyze the collected data.

Results: A total of 600 posts were initially evaluated, of which only 418 posts (69.67%) met the inclusion criteria. Images (79.7%) were the most common type of post. Hospitals (31.34%) and survivors/patients (18.9%) were the most common uploaders. There was a statistically significant difference in the quality (GQS) of posts uploaded by doctors, hospitals, healthcare organizations, patient survivors, and others (p <0.001).

Conclusions: There is a significant difference in the quality of posts uploaded by healthcare organizations compared to other groups. Government agencies and medical organizations impose tougher rules on the quality and trustworthiness of the type of healthcare-related information transmitted in order to minimize the distribution of low-quality and unreliable information.

## Introduction

Pneumonia is an infection of the lung parenchyma. It is mainly classified into community-acquired pneumonia, healthcare-acquired pneumonia, and aspiration pneumonia [1]. Pneumonia can be bacterial, viral, or fungal but bacterial pneumonia is more commonly encountered. *Streptococcus pneumonia* is the most encountered bacteria followed by *Klebsiella pneumoniae* and *Haemophilus influenzae* [1]. The most common causes of viral pneumonia include the influenza virus, respiratory syncytial virus (RSV), and recently severe acute respiratory syndrome coronavirus 2 (SARS‑CoV‑2) [1]. Although it can affect anyone, pneumonia is commonly seen in children aged ≤ two years and older adults ≥ 65 years [2]. Other risk factors include hospitalization, chronic obstructive pulmonary disease (COPD), smoking, and immunosuppression [2]. Signs and symptoms of pneumonia mainly include cough with greenish or yellow sputum, fever with chills and rigor, shortness of breath, pleuritic chest pain, and confusion seen especially in older people.

Even with emerging treatment options and vaccination facilities, pneumonia continues to be one of those diseases with a high global burden. Around 2.5 million people died from pneumonia in 2019. Almost a third of all victims were children younger than five years; it is the leading cause of death for children under five [3]. The most accurate test to diagnose pneumonia is CT as it provides detailed information about lung parenchyma and mediastinum [4]. The mainstay of pneumonia treatment is empiric antibiotics, and the choice of antibiotic depends on the setting in which the patient is treated [4]. Pneumonia is one among the vaccine-preventable infectious diseases and available vaccines include pneumococcal conjugate vaccine (PCV13, PCV15, and PCV20) and pneumococcal polysaccharide vaccine (PPSV23) [4].

Over the past few years, the internet and social media platforms have emerged as the predominant channels through which patients seek information about their medical conditions. As of 2020, 60% of the world’s population had access to the internet [5] and as of 2023, Instagram (Meta Platforms, Inc., Menlo Park, California, United States) boasts 2 million monthly active users. This makes it the fourth most used social media platform [6].

The objective of this study was to analyze the quality and reliability of Instagram posts about pneumonia. This study examines the characteristics of Instagram posts containing a hashtag related to pneumonia and evaluates the quality and reliability of the information presented regarding pneumonia, thereby contributing to the understanding of the potential significance of such content within the broader context of healthcare-related online discourse.

## Materials and methods

A cross-sectional observational study was conducted, wherein data was collected from Instagram over a three-day period from August 2-4, 2023. Each author was allotted one of the following hashtags: #pneumonia, #pneumoniasucks, #pneumoniatreatment, #pneumoniaawareness, #pneumoniaviral, #pneumoniaisnojoke. A premade questionnaire was used by the authors to collect data.

Each author analyzed the top 100 posts in each hashtag using the criteria that the posts are to be in the English language and should be relevant to the disease, i.e. pneumonia. The characteristics of posts that were collected were: type of post (i.e. image or video/reel), total number of audiences reached (i.e. number of likes, comments, followers), and type of uploader (i.e. doctor, hospital, healthcare organization, survivors/patients, or others). Each post was also analyzed regarding the type of information shared such as the description of symptoms, information about prevalence/incidence, information about cause/etiology, information about the diagnosis, information about vaccines/shots, information about treatment, information about mortality, information about rehabilitation, information about support groups, whether the post was a digitally created image/video, and whether the post has promotional content by any pharmaceutical company or doctors.

The quality and reliability of posts were then analyzed by the Global Quality Score (GQS) and DISCERN Reliability Score [7]. Statistical analysis was done using IBM SPSS Statistics for Windows, Version 21.0 (Released 2012; IBM Corp., Armonk, New York, United States.

## Results

For this study, each of the six authors analyzed 100 posts. Out of a total of 600 posts analyzed based on inclusion and exclusion criteria, 418 posts were included in the study. Table 1 describes the total posts evaluated and the number of posts included.

**Table 1:** Total Posts Evaluated and Included in the Study

S.no	Hashtag	Posts analyzed	Posts Included
1	#pneumonia	100	35
2	#pneumoniaawareness	100	75
3	#pneumoniaisnojoke	100	89
4	#pneumoniasucks	100	49
5	#pneumoniatreatment	100	70
6	#pneumoniaviral	100	100
	Total	600	418

Table 2 describes the characteristics of posts. The first characteristic evaluated was the type of the post, out of which the majority (79.7%) were images. The second characteristic was the number of audience reactions, in that the number of likes was 197,118, the number of comments was 1176, and the number of followers was 17,777,992. The third characteristic was the type of uploaders, with the highest (31.34%) being hospitals followed by survivors/patients (18.9%).

**Table 2:** Characteristics of Instagram* Posts about Pneumonia *Meta Platforms, Inc., Menlo Park, California, United States

Criteria	n (%)
Type of posts	
Image	333 (79.7%)
Video/reel	85 (20.3%)
Total audience reached	
Number of likes	197118
Number of comments	1176
Number of followers	17777992
Type of uploader	
Doctor	51 (12.2%)
Hospital	131 (31.34%)
Healthcare organization and health website	34 (8.13%)
Survivors and patients	79 (18.9%)
Others	123 (29.43%)

Table 3 describes the type of information shared in the posts. The majority (55.74%) of the information was shared in the form of a digitally created image/video. The majority (46.1%) of the posts were regarding the description of the disease, or explaining what it was.

**Table 3:** Type of Information shared in Instagram* Posts about Pneumonia *Meta Platforms, Inc., Menlo Park, California, United States

Information	n (%)
Description about the disease (Explaining what is it)	193 (46.17%)
Description of symptoms	189 (45.22%)
Information about prevalence/incidence	74 (17.7%)
Information about cause/etiology	140 (33.49%)
Information about diagnosis	121 (28.95%)
Information about vaccines/shots	94 (22.49%)
Information about treatment	83 (19.86%)
Information about mortality	107 (25.6%)
Information about rehabilitation	13 (3.11%)
Information about support groups	9 (2.15%)
Information about people/patients sharing their own experiences	58 (13.88%)
Information about parent sharing their experience with their family members	19 (4.55%)
Is it a digitally created image/video	233 (55.74%)
The post has promotional content by pharmaceutical companies or by doctors	108 (25.84%)

Table 4 describes the assessment of the quality and reliability of posts based on uploaders.

**Table 4:** Assessment of Quality and Reliability of Posts based on Uploader Values are written as median (Q1, Q3) where Q is quartile; p <0.05 is significant

	Doctor	Hospital	Healthcare organization and health website	Survivors/Patients	Others	P-value (Method: Kruskal-Wallis Test)
Global Quality Score	2 (2,3)	2 (2,3)	3 (2,4)	2 (1,2)	2 (1,3)	<0.001
Reliability score (DISCERN)	2 (2,3)	2 (2,3)	3 (2,4)	2 (1,2)	2 (2,3)	0.11

The median GQS for the posts uploaded by doctors was 2, hospitals 2, health care organizations and health websites 3, and survivors/patients 2. The difference was found to be statistically significant (p<0.05). The median DISCERN/reliability score for posts uploaded by doctors was 2, hospitals 2, health care organizations and health websites 3, and survivors/patients 2. The difference was not found to be statistically significant (p>0.05).

## Discussion

Our study analyzed 600 Instagram posts with a hashtag related to pneumonia and thereafter included 418 posts (69.67%) that were in the English language and disease-relevant. The posts included in the study had a very wide reach as evidenced by the number of followers of the posts’ authors, i.e., 17,777,992, and the number of people reacting to these posts in the form of likes i.e., 197,118.

Given the large reach of these posts, our study presented many valuable findings regarding information type and quality being presented by different types of uploaders to Instagram users. The majority of the posts were from hospitals (31.34%) with most of the information comprising an explanation of pneumonia (46.17%) followed by a description of the symptoms experienced in pneumonia (45.22%). The GQS and reliability score of posts uploaded by different authors were low, none of them exceeding the median value of 3. The difference between the GQS of different authors was statistically significant (p<0.001) whereas the reliability score difference was statistically insignificant (p=0.11).

Our study assessed the quality of posts based upon GQS from 1-5 with most of the posts being of low quality. In a similar study, assessing YouTube (Google LLC, Mountain View, California, United States) as a source of information on the link between coronavirus disease 2019 (COVID-19) and rheumatic disease, the quality of posts was based on GQSs of high, moderate, or low quality with most of the posts being high quality (41.4%), followed by low-quality posts (36.9%) [8]. In contrast, in our study, the majority of the posts were of low quality.

In our study, the highest GQSs and DISCERN scores were achieved by posts from healthcare organizations (GQS=3), whereas in a similar study, regarding COVID-19-related medical information on YouTube, the highest GQS and DISCERN scores were achieved by government-agency-generated videos (DISCERN 5.0, GQS 4.0) [9].

The posts analyzed in this study had 197,118 likes and the majority of the posts were uploaded by hospitals (31.34%). In a similar study regarding healthcare information on YouTube about pregnancy and COVID-19, the total number of likes of the videos included was 40,849 and the majority of the posts were uploaded by physicians (73%) [10]. Therefore, our study results were based on findings from a wider audience.

Limitations

As our study includes the posts analyzed during a three-day period, the number of likes, dislikes, and comments can be changed subsequently, differing from the current values. There is a probability of interobserver bias regarding GQS and DISCERN scores.

## Conclusions

Using Instagram hashtags, the study identified a low quality and reliability of informational posts regarding pneumonia. Given the large number of uploads on Instagram daily, it is notably challenging to ensure the quality and reliability of the information shared through this platform, as well as other social media sites. To minimize the dissemination of low-quality and unreliable information, it is recommended that institutions, such as medical organizations and government authorities, enforce stricter regulations on the quality and reliability of the type of information spread. Such initiatives will enhance patient education and the decision-making of the general public in the long term.
